# Assisted Reproductive Technology in China: Results Generated From Data Reporting System by CSRM From 2013 to 2016

**DOI:** 10.3389/fendo.2020.00458

**Published:** 2020-09-17

**Authors:** Linli Hu, Zhiqin Bu, Guoning Huang, Haixiang Sun, Chengyan Deng, Yingpu Sun

**Affiliations:** ^1^Henan Province Key Laboratory for Reproduction and Genetics, Reproductive Medical Center, The First Affiliated Hospital of Zhengzhou University, Zhengzhou, China; ^2^The 4th Branch of Chinese Society of Reproductive Medicine (CSRM), Beijing, China; ^3^Chongqing Key Laboratory of Human Embryo Engineering, Chongqing Reproductive and Genetics Institute, Chongqing Health Center for Women and Children, Chongqing, China; ^4^Reproductive Medicine Center, Drum Tower Hospital of Nanjing University, Nanjing, China; ^5^Department of Obstetrics and Gynecology, Peking Union Medical College Hospital (PUMCH), Beijing, China

**Keywords:** assisted reproductive technology, data reporting system, Chinese Society for Reproductive Medicine, pregnancy outcomes, CSRM

## Abstract

**Background:** What are the trends and figures in the treatments involving Assisted Reproductive Technology (ART) in mainland China?

**Method:** The Chinese Society of Reproductive Medicine (CSRM) retrospectively collected and analyzed data from 2013 to 2016 in 28 province of China by CSRM ART Data Reporting System.

**Results:** Among the 327 centers in China by December 2016, 133 centers reported 1,211,303 cycles and 470,725 infants in the 4 year period. Since 2013, the total number of frozen embryo transfer (FET) cycles, PGD/PGS cycle showed an increasing trend year by year. However, the number of donor sperm (DS) and donor egg (DE) cycles remained at a low level. Pregnancy outcomes such as implantation rate, pregnancy rate and delivery rate per embryo transfer cycles were stable in all types of ART, but decreased dramatically with increasing age. However, the average number of transferred embryos gradually decreased from 2013 to 2016, especially in PGD/PGS cycles. Thus, multiple pregnancy rate also decreased, it decreased significantly in PGD/PGS cycles from 30.5% in 2013 to only 1.7% in 2016.

**Conclusions:** The current study gives valuable information for both physicians and patients to know better about the outcome, as well as for administrators for policy development.

## Introduction

Ten years following the world's first baby was born as a result of *in vitro* fertilization (IVF) in the UK, the first IVF baby in China was born in 1988 (1). As use of advanced technologies to overcome infertility has increased, more and more infertility patients benefit from assisted reproductive technologies (ART). Various reporting systems for ART were developed. These include Centers for Disease Control and Prevention (CDC) report in the USA and European IVF Monitoring (EIM) Consortium on behalf of the European Society of Human Reproduction and Embryology (ESHRE) Program in Europe (2). The International Committee for Monitoring Assisted Reproductive Technologies (ICMART) has given reports of worldwide ART data from 2008 to 2010 (3).

In order to record the clinical outcomes and characteristics of ART and to help management, guide and develop Chinese ART, the Chinese Society of Reproductive Medicine (CSRM) implemented a registry report system for the data of assisted reproductive technology (ART) in 2015. Starting with the report for 2013, the CSRM has reported the clinical outcomes of ART. This is the first analysis of ART treatments based on data from the ART clinics in China that voluntarily provided data on procedures performed between January 2013 and December 2016.

## Materials and Methods

Data on ART were collected in 28 province of China by CSRM ART Data Reporting System which is the web-based data collection system (http://59.110.12.46/). Up to December 31, 2016, 327 reproductive centers were approved by National Health and Family Planning Commission of PRC (before March, 2018) or National Health Commission of PRC (after March, 2018) to conduct ART treatments. Since 2015, CSRM has requested that all members and other ART clinics to voluntarily register and report ART data, including IVF, intracyto plasmic sperm injection (ICSI), half ICSI and rescue ICSI(IVF+ICSI), frozen embryo transfer(FET), donor egg(DE), donor sperm(DS), Pre-implantation Genetic Diagnosis/Screening (PGD/PGS). However, IUI cycles using husband semen (IUI-H) and donor semen (IUI-D) were not included. All data were retrospectively collected.

The same data forms were made available to the each participating center. Data collected were directly submitted to CSRM online system that can automatically calculate all the results. Then each center reporting data can view the characteristics and treatment outcomes of registered cycles from the Center, the province and the country.

The definition of terms is derived from the International Committee for Monitoring Assisted Reproductive Technology (ICMART) and World Health Organization glossary of ART terminology (4).

## Results

### Participation and Number of Treatment Cycles per Technique and Availability

The present report includes data from 31 of 34 Chinese provincial administrative regions. The total number of registered cycles showed an increasing trend from 2013 to 2016. There were 235,739 cycles and 94,766 infants, 287,424 cycles and 115,191 infants, 318,045 cycles and 125,118 infants, 370,095 cycles and 135,650 infants reported from 2013 to 2016, respectively. In total, there were 1,211,303 cycles resulting in delivery of 470,725 infants (Table 1). Among the total of 370,095 fresh cycles reported in 2016, 147,810 were IVF (74.0%) and 52,298 were ICSI (26.0%) (Table 1). Since 2013, the ratio of IVF to ICSI cycles has been similar. The total number of FET cycles from 2013 to 2016 was 81,929, 106,419, 122,312, and 151,889, respectively, that showed an increasing trend (Table 1). In the PGD/PGS cycle, there is the same upward trend, and the number of cycles from 2013 to 2016 is 729, 1,165, 1,890, 2,779 (Table 1). The number of DS and DE cycles remained at a low level that were 2 812(1.2%) and 446(0.2%), 4 168(1.5%) and 448(0.2%), 4,923(1.5%) and 423(0.1%), 4,737(1.3%) and 325(0.1%) from 2013 to 2016, respectively.

**Table 1:** Number of clinics, cycles, and ART infants of different technologies in 2013–2016.

**Type of cycle**	**2013**			**2014**			**2015**			**2016**			**Total**	
	**Clinics**	**Cycles**	**Infants**	**Clinics**	**Cycles**	**Infants**	**Clinics**	**Cycles**	**Infants**	**Clinics**	**Cycles**	**Infants**	**Cycles**	**Infants**
IVF	115	102,115	36,850	125	120,029	41,767	131	127,328	40,461	133	148,710	40,959	549,570	160,037
ICSI	114	38,243	13,558	123	45,380	14,553	128	49,801	14,616	130	52,298	13,625	207,316	56,352
IVF+ICSI	114	9,465	3,739	123	9,815	3,390	128	11,368	3,615	130	10,257	3,016	40,905	13,760
PGD/PGS	27	729	248	26	1,165	387	26	1,890	570	24	2,779	796	6,563	2,001
FET	115	81,929	39,102	125	106,419	53,295	131	122,312	64,059	133	151,889	75,452	462,549	231,908
DS	114	2,812	1,114	124	4,168	1,646	128	4,923	1,604	130	4,737	1,661	16,640	6,025
DE	114	446	155	123	448	153	128	423	193	130	325	141	1,642	642
Total		235,739	94,766		287,424	115,191		318,045	125,118		370,995	135,650	12,12,203	470,725

*DS, IVF/ICSI cycles using donor sperm; DE, IVF/ICSI cycles using donor egg*.

### Outcomes of Fresh and Frozen Non-donor Cycles

The characteristics and treatment outcomes of the fresh cycles are shown in Table 2. Even if the total number of fresh cycles showed an increasing trend, the proportion of IVF to ICSI cycles has stabilized since 2013 to 2016 and 147,810 initiated IVF (74.0%) and 52,298 initiated ICSI (26.0%) cycles in 2016.

**Table 2:** Results after fresh and frozen non-donor cycles in 2013–2016.

**Type of cycle**	**2013**						**Total**	**2014**						**Total**	**2015**						**Total**	**2016**						**Total**
	** <35**	**35-37**	**38-40**	**41-42**	**43-44**	**>44**		** <35**	**35-37**	**38-40**	**41-42**	**43-44**	**>44**		** <35**	**35-37**	**38-40**	**41-42**	**43-44**	**>44**		** <35**	**35-37**	**38-40**	**41-42**	**43-44**	**>44**	
**IVF**																												
Initiated cycles	7,2372	14,650	9,353	3,264	1,602	874	10,2115	84,547	17,335	10,814	4,036	2,070	1,227	120,029	90,545	18,090	10,516	4,425	2,263	1,489	127,328	98,951	20,179	14,496	6,935	4,409	2840	147,810
Aspirated cycles	65,940	13,240	8,340	2,762	1,303	646	92,231	77,183	15,559	9,613	3,432	1,692	936	108,415	80,472	15,628	9,011	3,654	1,714	1,064	111,543	89,882	18,576	12,634	5,785	3,506	2193	132,576
Transfer cycles	50,541	10,761	6,633	2,138	905	424	71,402	55,388	11,776	6,926	2,348	1,001	490	77,929	52,946	10,985	6,041	2,171	889	463	73,495	50,986	11,314	7,091	2,900	1,409	715	74,415
Average number of transferred embryos	1.96	2.18	2.24	2.17	2.10	1.90	2.03	1.92	2.15	2.12	2.16	2.05	1.91	1.98	1.88	2.00	2.03	2.14	2.06	1.79	1.92	1.86	1.89	1.92	1.94	1.94	1.85	1.87
Implantation rate (%)	37.9	28.0	20.7	13.0	7.8	5.2	33.2	39.5	29.1	22.7	12.7	7.5	4.3	34.7	40.9	31.5	22.2	12.9	7.7	3.3	36.2	41.8	33.6	23.8	14.7	8.4	5.1	36.7
PR per aspiration (%)	41.1	37.5	29.2	18.7	9.7	5.9	38.2	40.0	37.0	26.1	15.8	8.2	3.5	36.7	37.4	33.6	24.8	14.0	7.5	2.1	34.3	32.8	30.1	21.1	12.5	6.0	2.9	29.2
PR per transfer (%)	53.6	46.2	36.7	24.1	14.0	9.0	49.3	55.7	48.8	36.3	23.1	13.8	6.7	51.1	56.8	47.8	37.0	23.6	14.4	4.8	52.0	57.8	49.5	37.7	24.9	14.8	8.8	52.1
DR per aspiration (%)	34.2	28.9	20.0	10.4	4.8	1.9	30.8	33.2	28.2	18.1	9.0	3.8	1.6	29.7	30.8	26.2	17.2	8.2	3.0	0.8	27.6	28.0	23.5	14.7	6.7	2.7	1.0	24.1
DR per transfer (%)	44.6	35.5	25.1	13.4	6.9	2.8	39.7	46.3	37.3	25.2	13.2	6.4	3.1	41.3	46.8	37.2	25.7	13.7	5.7	1.7	41.9	49.4	38.6	26.2	13.4	6.7	3.1	42.9
**ICSI**																												
Initiated cycles	27,422	5,005	3,320	1,326	754	416	3,8243	31,821	6,054	4,101	1,757	1,007	640	45,380	35,422	6,435	4,220	1,895	1,109	720	49,801	35,310	6,827	4,964	2,405	1,637	1,155	52,298
Aspirated cycles	25,238	4,566	3,026	1,152	622	309	34,913	2,9358	5,522	3,696	1,512	867	499	41,454	32,039	5,720	3,729	1,590	929	536	44,543	32,037	5,978	4,304	1,979	1,321	932	46,551
Transfer cycles	19,016	3,549	2,247	795	368	174	26,149	20,003	3,955	2,438	903	430	210	27,939	19,655	3,590	2,170	816	402	189	26,822	17,798	3,346	2,247	879	478	261	25,009
Average number of transferred embryos	1.99	2.21	2.21	2.09	2.01	1.86	2.04	1.96	2.21	2.12	2.03	1.99	1.95	2.01	1.91	2.08	2.01	1.88	1.95	1.83	1.94	1.85	1.90	1.92	1.93	1.91	1.90	1.87
Implantation rate (%)	36.1	26.6	19.7	10.9	6.7	3.1	31.8	38.1	25.6	18.2	12.5	5.7	2.9	32.8	39.6	28.3	20.8	13.2	5.7	2.6	34.9	40.7	30.2	21.8	12.4	6.1	3.8	35.4
PR per aspiration (%)	39.7	34.2	25.3	13.3	6.9	2.9	35.9	37.3	31.2	20.9	14.2	5.1	2.2	33.1	34.2	28.3	19.6	11.3	4.5	1.5	30.4	30.8	25.4	18.2	9.5	3.8	1.8	26.7
PR per transfer (%)	52.6	44.0	34.1	19.2	11.7	5.2	48.0	54.7	43.6	31.7	23.7	10.2	5.2	49.1	55.7	45.1	33.6	22.1	10.4	4.2	50.4	55.5	45.4	34.9	21.4	10.5	6.5	49.7
DR per aspiration (%)	33.9	27.0	17.7	7.9	2.9	1.3	29.9	31.7	23.9	14.7	7.6	2.9	1.6	27.3	29.4	21.5	14.3	6.0	2.0	0.6	25.4	27.2	20.0	13.0	4.6	1.7	0.5	22.7
DR per transfer (%)	45.0	34.7	23.9	11.4	4.9	2.3	39.9	46.5	33.3	22.3	12.7	5.8	3.8	40.5	48.0	34.2	24.6	11.6	4.7	1.6	42.2	48.9	35.7	24.8	10.5	4.6	1.9	42.3
**FET**																												
Transfer cycles	61,293	11,328	6,089	1,948	870	401	81,929	78,926	14,496	8,253	2,670	1,391	683	106,419	88,068	17,736	9963	3,648	1,829	1,068	12,2312	107,446	2,1678	12,584	5,291	3,052	1,838	151,889
Average number of transferred embryos	2.03	2.04	2.06	2.08	2.00	2.01	2.04	1.99	2.03	2.04	2.04	2.08	1.94	2.00	1.92	1.94	1.96	1.99	1.97	1.97	1.93	1.80	1.78	1.79	1.84	1.85	1.81	1.80
Implantation rate (%)	33.9	28.4	20.3	14.1	10.5	9.4	31.2	37.0	28.5	21.6	14.8	10.0	6.0	33.5	39.6	31.1	25.1	16.4	10.7	7.4	35.7	40.3	32.0	25.0	16.5	10.0	5.9	35.9
PR per transfer (%)	50.6	42.6	33.8	24.3	18.0	15.7	47.1	53.1	44.8	36.3	26.3	18.5	9.4	49.3	56.2	47.3	39.6	26.0	16.6	12.7	51.7	55.4	46.0	37.3	26.4	17.1	9.2	50.2
DR per transfer (%)	41.5	32.3	22.9	12.7	9.3	8.0	37.7	43.7	33.5	24.9	15.8	9.5	5.0	39.5	45.7	36.3	28.3	16.4	8.6	5.8	41.1	45.2	35.0	25.5	14.9	9.0	4.7	39.8

*PR, Clinical pregnancy rate; DR, Delivery rate*.

Clinical outcomes varied among patients with different female ages. Implantation rate, pregnancy rate, as well as delivery rate, decreased dramatically with increasing age in all ART cycles. For instance, in 2016 IVF delivery rate per embryo transfer in <35, 35–37, 38–40, 41–42, 43–44, and >44 years old were 49.4, 38.6, 26.2, 13.4, 6.7, and 3.1%, respectively. ICSI delivery rate per embryo transfer in <35, 35–37, 38–40, 41–42, 43–44, and >44 years old were 48.9, 35.7, 24.8, 10.5, 4.6, and 1.9%, respectively. Moreover, FET delivery rate per embryo transfer in these 6 groups were 45.2, 35.0, 25.5, 14.9, 9.0, and 4.7%, respectively.

The IVF average number of transferred embryos gradually decreased from 2.03 in 2013 to 1.87 in 2016, and there are the same decreasing trend in ICSI and FET. However, from 2013 to 2016, the implantation rate of IVF and ICSI increased from 33.2 to 36.7% and from 31.8 to 35.4%. In terms of frozen cycles, the implantation rate were increased from 31.2 to 35.9 % (Table 2).

The pregnancy rate (PR) per oocyte retrieval for IVF and ICSI from 2013 to 2016 significantly decreased from 38.2 to 29.2% and from 35.9 to 26.7%, respectively. However, during this time period the PR per embryo transfer for IVF and ICSI seems to have slightly increased from 49.3 to 52.1% and from 48.0 to 49.7%, respectively. The delivery rate (DR) from 2013 to 2016 have the same trend with PR. DR per oocyte retrieval for IVF and ICSI decreased from 30.8 to 24.1% and from 29.9 to 22.7%, respectively. DR per embryo transfer for IVF and ICSI increased from 39.7 to 42.9% and from 39.9 to 42.3%. In FET cycles the PR and DR per transfer were 50.2 and 39.8% in 2016 that were stable from 2013 to 2016.

### Outcomes of Fresh/Frozen Donor Cycles and PGD/PGS Cycles

Numbers of DS cycles have been increased year by year from 2013 (2,812 cycles) to 2016 (4,737 cycles). However, the number of DE cycles has been stable in the four year period. As for pregnancy rate and delivery rate per transfer cycle, they are similar with that in fresh IVF/ICSI cycles (Table 3).

**Table 3:** Results after PGD/PGS, DS, and DE in 2013–2016.

	**2013**	**2014**	**2015**	**2016**	**Total**
**DS**					
Initiated cycles	2,812	4,168	4,923	4,737	16,640
Transfer cycles	1,910	2,444	2,442	2,362	9,158
Average transferred embryos	1.92	1.91	1.90	1.84	1.89
Implantation rate (%)	33.9	40.8	31.7	44.1	37.7
PR per transfer (%)	48.6	57.1	59.1	62.4	57.2
DR per transfer (%)	32.6	49.2	50.1	54.0	47.2
**DE**					
Transfer cycles^a^	297	300	330	252	1179
Average transferred embryos	2.04	1.91	1.96	1.96	1.97
Implantation rate (%)	40.9	35.6	37.0	35.8	37.4
PR per transfer (%)	47.5	52.3	52.1	52.8	51.1
DR per transfer (%)	38.7	41.7	45.8	43.7	42.5
**PGD/PGS**					
Transfer cycles^b^	587	809	1,189	1,765	4,350
Average transferred embryos	1.69	1.57	1.20	1.00	1.25
Implantation rate (%)	55.9	59.8	59.3	53.2	56.5
PR per transfer (%)	43.6	48.7	52.7	53.1	50.9
DR per transfer (%)	34.2	39.3	43.6	44.6	41.9

a *Cycles transferred using thawed embryos from fresh DE cycles*.

b *Total cycles transferred using fresh or thawed embryos from fresh PGD/PGS cycles*.

As for PGD/PGS, treatment cycles also increased dramatically from 2013 to 2016, which is consist with the increase of centers permitted to perform PGD/PGS treatment (Table 3). Even pregnancy outcomes regarding to implantation rate, pregnancy rate, as well as delivery rate, have been stable from 2013 to 2016, the number of embryos transferred per cycle decreased significantly from 1.69 in 2013, to 1.00 in 2016, which indicates that all center followed a strict one-embryo-transfer policy.

### Multiple Pregnancy, Miscarriage, and Birth Defects Rate in Art

Multiple pregnancy, miscarriage and birth defects rate in all kinds of ART were clearly shown in Table 4. In total, multiple pregnancy rate was more than 30% in all IVF/ICSI cycles. Interestingly, multiple pregnancy rate was only 12.4% in PGD/PGS cycles. Even multiple pregnancy rate decreased from 2013 to 2016 in all cycles, it decreased significantly in PGD/PGS cycles from 30.5% in 2013 to only 1.7% in 2016.

**Table 4:** Multiple pregnancy, miscarriage and birth defects rate of all kinds of ART in 2013–2016.

	**2013**			**2014**			**2015**			**2016**			**Total**		
	**MPR (%)**	**MR (%)**	**BDR (%)**	**MPR (%)**	**MR (%)**	**BDR (%)**	**MPR (%)**	**MR (%)**	**BDR (%)**	**MPR (%)**	**MR (%)**	**BDR (%)**	**MPR (%)**	**MR (%)**	**BDR (%)**
IVF	34.2	14.7	0.9	33.8	14.4	0.8	33.4	14.1	0.9	33.0	15.0	0.9	33.6	14.5	0.9
ICSI	33.8	13.4	0.9	33.2	13.9	0.9	32.7	14.1	1.0	32.0	14.1	1.0	32.9	13.9	0.9
PGD/PGS	30.5	19.5	0.4	24.4	16.5	0.8	13.4	17.2	0.5	1.7	13.6	1.4	12.4	15.8	1.0
FET	32.3	16.0	0.8	31.8	16.4	0.8	31.6	16.0	0.7	29.4	16.8	0.9	31.0	16.4	0.8
DS	33.9	6.6	1.3	37.8	11.9	0.7	32.6	11.9	0.7	31.5	11.7	1.2	33.9	10.9	0.9
DE	42.6	13.5	0.6	28.0	14.0	0.7	33.7	12.2	0.5	31.6	11.3	1.4	33.8	12.8	1.1

*DS, IVF/ICSI cycles using donor sperm; DE, IVF/ICSI cycles using donor egg*.

*MPR, multiple pregnancy rate, calculated using the number of multiple pregnancy cycles divided by the number of pregnancy*.

*MR, miscarriage rate, calculated using the number of miscarriage cycles divided by the number of pregnancy*.

*BDR, birth defects rate, calculated using the number of infants with birth defects divided by the total number of infants*.

As for miscarriage rate and birth defects rate, they were stable from 2013 to 2016, and comparable between different types of ART treatment. However, compared with those in non-donor cycles, miscarriage rate tended to be lower in DE and DS cycles.

## Discussion

Infertility is defined as the inability to conceive after 12 months of unprotected intercourse. Infertility affects 10–15% of couples (5). This makes it one of the most common diseases for couples in fertile age. Since last century, infertility has been proposed to be treated by assisted reproductive technology (ART), which refers to all methods involving the manipulation of eggs, sperm or embryos *in vitro* to achieve the purpose of pregnancy. Nowadays, ART has evolved several medical interventions mainly include IUI, IVF/ICSI, PGD/PGS, and their derivatives, and resulted in the birth of more than 6 million children worldwide (6, 7).

The first fresh IVF baby in mainland China was born in Peking University Third Hospital in 1988. During the past 30 years, most reproductive centers in China can perform routine IVF/ICSI, and can maintain a stable pregnancy rate. However, as a country with the world biggest population, hundreds of thousands ART babies were born each year in China, yet the detailed information of these treatment cycles were unpublished (8, 9).

The CSRM was established as a branch of the Chinese Medical Association (CMA) in 2005, and then the sub-societies were organized in provinces, autonomous regions and municipalities. Its major aim was to help management, guide, and develop Chinese ART, train new medical staff and incorporate cooperation with international reproductive societies or associations. The data collection of this report was initiated and supervised by sub- society of CCRM in Henan province. According to the statistics of the National Health Commission, up to December 2016, there were as many as 327 reproductive centers conducting assisted reproductive technology. In order to standardize and promote the development of assisted reproductive technology in China, and to provide quality control for reproductive centers; meanwhile, in order to provide data support for health administrative departments, and to facilitate the verification and admission review of various centers, CSRM developed a data reporting system for human assisted reproduction technology in China in 2015.

In other counties and regions such as Europe, America, and Australia, a complete ART data reporting system has been formed in the early 1990s. At present, about 97.0% of centers in the United States report data every year. However, the proration of centers reporting data in China was only 40.67% (133/327) up to 2016. Although the number of data reporting centers not huge, with the continuous efforts of CSRM, the number of centers participating in data reporting has increased year by year. From 2013 to 2016, the number of reported cycles had been increased to 1 211 303 cycles, and the number of newborns to 470,725. However, we should be caution that due to the small number of centers reporting data, the collected data may have some inevitable bias. In addition, the processing of missing data in some centers still requires more experience to handle.

First, as for number of treatment cycles, the number of ART cycles is increasing year by year in the past 4 years. Meanwhile, with the increased frozen embryos resulted from oocyte retrieval, the proportion of frozen-thawed transfers in IVF cycles has also been increased dramatically. Although ART is an effective method for infertility treatment, physicians should be cautioned that the indications must be strictly controlled. Compared with IUI or natural conception, IVF treatment is more invasive. Although the increase in the number of IVF cycles may be related to the increased demand of infertility patients, we should also consider the possibility of providers being biased for IVF. On the other hand, with the continuous increase of the fresh oocyte retrieval cycle, it is understandable that the proportion of frozen embryo transfer increases. Since frozen embryo transfer not only increases the cumulative pregnancy rate of IVF, but also effectively reduces the risk of complications such as OHSS. Therefore, it is widely used in clinical practice, which also suggests that we need to pay more attention to the safety of babies born after frozen embryo transfers in future work. In addition, in terms of the number of IVF/ICSI cycles, ICSI ratio in China has always been lower than that in other countries. This is because in China ICSI is only used in men with severe oligospermia, azoospermia, or PGD/PGS cycles (10–12).

Secondly, in terms of pregnancy outcomes, although the pregnancy rate and delivery rate of all types of ART have both increased over the past 4 years, they have increased very slowly. In recent years, the ovarian stimulation protocols China have been dominated by GnRH agonist, and the type of embryos transferred is still cleavage-stage. Although the dosage and types of GnRH agonist have been slightly changed, this stability of ovarian stimulation protocols and embryo transfer strategy is the cause of stable pregnancy outcomes (13).

The number of sperm donation/egg donation cycles, as well as PGD cycles is relatively small in China. Because gamete donation is strictly regulated; reproductive centers must obtain special licenses. According to regulations, the donors of gametes must be voluntary, gratuitous and anonymous. However, due to the limitation of egg frozen technology, and special policies of egg donation in China, the number of egg donation cycles has been very stable in recent years (14, 15).

Multiple pregnancy is defined as having two or more fetuses in the uterine cavity of a pregnancy at the same time. The incidence of multiple pregnancies in natural pregnancy is very low, and is related to factors such as race, age and genetics. As multiple pregnancy may bring serve medical complications to both mothers and infants, the incidence should be strictly controlled. During assisted reproductive technology treatment, the incidence of multiple pregnancy is strongly correlated with the number of embryos transferred. From data in the current study, we can see that the average number of embryos transferred in recent years is around 2.0, so the incidence of multiple pregnancy is more than 30%, which is higher than that in other regions. However, we are glad to see that in the PGD cycle, the number of embryos transferred in recent years has been strictly controlled from 1.69 in 2013 to 1.0 in 2016, so the incidence of multiple pregnancy has dropped from 30.5% in 2013 to 1.7% in 2016. This also reminds us that in the future, selective single embryo transfer should be advocated in all reproductive centers.

Even as the first comprehensive ART data set from 133 centers in mainland China, there are some limitations in our data analysis. First, although there are a large number of centers in China, the proportion of data reported centers is low; thus there may be some bias in the data. Second, the quality of the reported data is not well-controlled, since errors may happen during the upload process. In addition, some other important parameters during the ART process, such as body mass index, cleavage or blastocyst stage embryo transfer, assisted hatching, etc., are needed to be further collected. Finally, because data are from multicenter, there is heterogeneity in the terms of definition and operation, which may impact on the quality of the final data. Since data registry report system is developed and designed by CSRM, and data are voluntarily reported by the centers included, we will continue to improve this system, and believe that more valuable data will be available soon.

In summary, the present analysis gives the status of ART for the period 2013–2016 in mainland China. It gives valuable information for both physicians and patients to know better about the outcome, as well as for administrators for policy development.

## Data Availability Statement

The raw data supporting the conclusions of this article will be made available by the authors, without undue reservation.

## Ethics Statement

Ethical review and approval was not required for the study on human participants in accordance with the local legislation and institutional requirements. Written informed consent for participation was not required for this study in accordance with the national legislation and the institutional requirements. Written informed consent was obtained from the individual(s) for the publication of any potentially identifiable images or data included in this article.

## Author Contributions

All authors listed have made a substantial, direct and intellectual contribution to the work, and approved it for publication.

## Conflict of Interest

The authors declare that the research was conducted in the absence of any commercial or financial relationships that could be construed as a potential conflict of interest.
